# Supplementary material for: Are carbon emissions trading and green financial instruments synergistic? -Comprehensive quantitative research based on content analysis
Source: PLoS One. 2024 Mar 7;19(3):e0298601. doi: 10.1371/journal.pone.0298601 (PMC10919601; doi:10.1371/journal.pone.0298601)
Supplement: S1 Data — (ZIP) [file pone.0298601.s001.zip › Data/地级市Z_F工作报告.zip!/╕ú╜¿/╒─╓▌╩╨/└┤╫╘╣1⁄2╓┌║┼í╛┬φ┐╦╩2╛▌═°í┐_2016.docx]

各位代表：

　　现在，我代表漳州市人民政府，向大会报告政府工作，请予审议，并请市政协委员和其他列席人员提出意见。

一、2015年和“十二五”时期工作回顾

2015年，在省委、省政府和市委的正确领导下，在市人大及其常委会和市政协的监督支持下，我市各级政府全面贯彻党的十八大和十八届三中、四中、五中全会精神，深入贯彻习近平总书记系列重要讲话精神和对福建工作的重要指示，认真落实市委十届十次、十一次全会精神，经济社会发展取得新成效。预计全市生产总值2766.6亿元，增长11.0%；一般公共预算总收入274.8亿元，增长4.14%，地方一般公共预算收入179.1亿元，增长5.98%；规模工业增加值1258.6亿元，增长10.6%；全社会固定资产投资2573.7亿元，增长20.6%；外贸出口463.6亿元，下降7.2%；实际利用外资10.85亿美元，增长7.2%；社会消费品零售总额增长12.2%；居民消费价格总水平上涨1.6%；城镇居民人均可支配收入28130元，增长9.3%；农民人均可支配收入13890元，增长9.5%；城镇登记失业率2.05%；人口自然增长率10.39‰。年度节能减排任务全面完成。

一年来的主要工作和成效是：

（一）应对下行压力，经济保持平稳增长。扎实开展“三比一看”“三个年”和“大干6个月”活动，持续追问题、解难题、促发展，出台扶持规模工业企业、外贸转型升级、建筑业发展、商贸流通业发展、电商与快递协同发展等惠企政策，

经济运行稳中有进，全市GDP增幅居设区市首位，比全省高2个百分点。开展“政策不睡觉、落地见成效”活动，累计减免各种企业税费34.25亿元；累计发放应急周转金67.2亿元，有效化解企业信贷风险。强化投资拉动，八大重点领域项目完成投资2500亿元，其中在建重点项目完成投资1251亿元，新开工亿元以上项目224个，总投资850亿元；完成城乡基础设施“六项提升工程”投资500亿元，完成电网建设投资23亿元，枋洋水利枢纽项目按计划推进。强化要素保障，成立用地保障工作领导小组，完成土地征迁2.1万亩，清理闲置土地1.6万亩。启动小微企业贷款保证保险工作，金融机构各项贷款余额1906.4亿元，增长16.3%，增幅居全省首位。列入国家专项建设基金项目53个,争取建设资金23.8亿元。新增上市企业2家、新三板挂牌7家。

（二）推进转型升级，产业实力得到提升。工业支撑持续增强,设立工业产业发展基金，实施产业龙头促进计划，45家省市级龙头企业产值增长11.6%，新增亿元企业159家，新增规模工业企业139家。完成技改投资570亿元，增长26.4%。推广“数控一代”机械产品，69家企业列入省级两化融合项目，5家省级众创空间投入运营，联东U谷创业创新基地获评全国青年创业示范区；新增省级企业工程技术研究中心和重点实验室6家、高新技术企业38家、专利授权3200项。第三产业加快发展，预计增加值增长14.7%，位居全省首位。四大电商平台上线运行，6个县（市）列入国家级、省级农村电子商务示范区。旅游业接待游客人数、旅游总收入分别增长15.2%和17.9%。打通商品房和保障性住房转换通道，全市商品房销售面积增长16.2%。现代农业提质增效，全市整体获批国家现代农业示范区，新增国家级出口食品农产品质量安全示范区1个、设施农业1.9万亩，新认证无公害、绿色和有机产品87个，10个产品入选全国名优新农产品名录，新增25艘远洋渔船。

（三）打造发展平台，重点区域加快突破。科学编制厦门港南岸新城核心区概念性城市设计、漳州高新区总体规划及产业发展规划、环东山岛经济区域协调发展规划，完善提升古雷石化基地发展规划，闽粤经济合作区发展规划获批实施。实施四大增长极项目222个，完成投资612亿元，占全市重点项目投资48.9%。古雷炼化一体化项目正式奠基，LNG接收站开工建设，漳州核电、古雷华能热电等重大项目前期工作取得明显进展；古雷整岛搬迁和征地清海工作基本完成。市级项目审批权限全部下放环东山岛经济区域，漳州高新区管理体制整合到位。漳州深化两岸产业合作示范区和古雷深化两岸石化产业合作示范区列入省委“十三五”规划建议和省“十三五”《纲要》，古雷综合保税区、台商投资区保税物流中心（B型）报批工作正在加紧推进。

（四）突破关键环节，改革开放纵深拓展。十大类61项改革任务进展顺利。完成工商、质监和食品药品管理体制改革，政府部门机构改革顺利完成，事业单位分类改革稳步推进。权力清单、责任清单、公共服务清单公布运行，保留行政审批事项118项、公共服务事项136项，在全省率先取消非行政许可审批事项，变行政审批为行政服务。居民生活用水、用电、用气全面实施阶梯价格制度，完善全口径预决算制度，商事登记制度改革扎实推进，出台加快开发区发展10条措施。市属重点国有企业完成资产整合，资产总额、营业收入分别增长29.6%、7.9%。新批千万美元以上外资项目数、注册合同外资额分别增长23.1%、35.1%，其中台资项目数增长144.4%。积极打造“海丝”重要节点城市，与东盟贸易额11.9亿美元，实际利用东盟资金增长41.6%。成功举办第七届海峡两岸农博会·第十七届海峡两岸花博会。在全省率先复制推广自贸区关检政策，关检“三个一”等便利通关模式全面推行。厦漳泉同城化步伐加快，对口支援、山海协作、援藏援疆援宁等工作有效开展。

（五）凸显闽南特色，城乡面貌持续改观。荣获第四届全国文明城市称号。启动“多规合一”试点工作，完成中心城区8个专项规划。实施中心城区城建项目465个，完成市政提升“五千工程”968公里，市区河道清淤40公里，新建改造城市道路216公里、地下管道（渠）639公里，新增绿道175公里、公园绿地1980亩、公共停车位1920个、公交车106辆。市区“五景”、闽南水乡示范段、滨江生态公园天宝段建设全面启动，古城保护建设（一期）进展顺利；闽南文化生态博览城核心区加快建设，闽南文化生态产业走廊荣获“全国生态文化示范基地”称号，西环城路综合景观工程获“中国宜居环境范例奖”。拆除“两违”770万平方米、河砂整治成果巩固扩大。漳浦、东山列入省级新型城镇化试点，角美、杜浔（古雷）列入省级小城市培育试点。农村建设“双百”行动深入开展，完成200个“富美乡村”规划，4个村入选首批国家级乡村旅游示范村，5个村入选国家旅游扶贫试点村。

（六）突出环境整治，生态建设力度加大。全面启动“七个五”生态建设行动计划，强力推进“三铁”治污，龙海、芗城、龙文全面完成2208家胶合板污染企业整治；淘汰黄标车6489辆。关闭拆除禁养区猪场15805家，削减生猪75.6万头，九龙江流域（漳州段）Ⅲ类水质达标率92.3%，市、县饮用水源水质达标率分别为100%、99.7%。新改扩建城镇污水处理厂27个，国家减排重点项目东墩、山格污水处理厂建成试运行，新建污水配套管网242公里，完成重点行业脱硫脱硝工作。划定林业六条生态红线，完成造林绿化20.97万亩，治理水土流失38.4万亩。荣获“国家森林城市”“省级生态市”称号。

（七）共享发展成果，民生事业持续改善。18件为民办实事项目全面完成，3.5万人实现脱贫。“造福工程”完成搬迁7830户，解决农村12.1万人饮用水安全问题。新开工建设中小学发展项目14.5万平方米，新建公办幼儿园17所，新增中小学、幼儿园学位18288个。新建续建卫生项目318个，新增病床位1531张、卫技人员3516名，培训全科医生和乡村医生4337名，医疗卫生事业三年行动计划超额完成。医疗改革稳妥推进，全市所有公立医院实现药品、耗材零差率销售，开展家庭医生签约服务试点工作，实现新农合、城镇居民医保“二保合一”。新农合筹资水平、农村低保标准、城乡居民基本医疗保险政府补助标准年人均分别提高到380元、2300元、380元，在全省率先开展新农合大病补充商业保险。获评“全国创建幸福家庭示范市”。“五馆一歌”建设全面推进，“台湾路—香港路街区”入选首批中国历史文化街区，圆满完成首届全国青运会三个承办比赛项目，新建全民健身场（所）58处。新增城镇就业5.13万人，转移农村劳动力7.25万人。新开工建设保障性安居工程26331套，基本建成30227套。顺利完成村级组织换届选举工作。“平安漳州”“法治漳州”建设持续深化，群众安全感满意度保持在97%以上，圆满完成“六五”普法任务。军政军民团结巩固发展。社会福利、社会救助、社会慈善、苏区老区等工作有效开展。市档案馆被确定为“全国中小学档案教育社会实践基地”。妇女儿童、青少年、民族宗教、广播电视、新闻出版、社会科学、地方志、气象、防灾减灾、残疾人、红十字会、工会、人防、物价等工作都取得新的进步。

（八）践行三严三实，自身建设进一步加强。深入开展“三严三实”专题教育，进一步弘扬谷文昌精神，认真贯彻中央“八项规定”精神，“四风”问题得到明显遏制。精简会议，减少发文，全市政府系统“三公”经费财政拨款支出下降15.17%。深化机关效能建设，强化“马上就办”，大力整治“庸懒散拖”。行政监察、审计监督等工作力度持续加大，政务公开和政府信息公开有序推进。认真执行人大决定决议，自觉接受人大及其常委会监督、政协监督和社会舆论监督，办理市人大代表建议171件、市政协提案316件，办结率100%。政府与政协、法院、工会互动联系机制不断完善，各民主党派参政议政富有成效。

2015年工作任务的完成，标志着“十二五”规划的顺利收官。

五年来，综合实力稳步增强。预计地区生产总值净增1335.9亿元、年均增长12.2%；人均地区生产总值55552元、年均增长11.4%。地方公共财政收入提前实现比2010年翻一番，全社会固定资产投资五年累计9072.67亿元，一批重大项目建成投用。

五年来，发展方式加快转变。三次产业结构由17.8∶45.6∶36.6优化为13.4：48.6：38.0。2015年规模工业总产值和规模工业增加值分别是2010年的2.14倍和2.12倍，第三产业增加值年均增长11.1%。产值超50亿元工业企业从2家增加到8家。高新技术产业产值、全社会研发投入分别是2010年的2倍和2.5倍；预计万元GDP能耗比2010年下降16%，完成节能减排任务。森林覆盖率从59.74%提高到63.58%。

五年来，基础设施持续完善。新增铁路营运里程211公里、公路通车里程1581公里、高速公路通车里程348公里，实现县县通高速、镇镇通干线、村村通客车；港口泊位货物年通过能力从2278万吨提高到4599万吨。电网建设累计投资87.9亿元，增长1.6倍。

五年来，人民生活不断改善。城乡居民人均可支配收入年均分别增长11.4%和12.4%。累计新增城镇就业人员26.7万人、转移农村劳动力36万人。“双高普九”全面实现，城乡居民社会养老保险制度实现一体化，基本医疗保险制度实现全覆盖，工伤、生育保险制度覆盖所有职业人群，城乡低保实现应保尽保。每千人口病床位数和卫技人员数分别从2.28张和2.45人提高到4.11张和4.52人。保障性安居工程超额完成省下达任务。累计投入扶贫开发资金15.6亿元，减少贫困人口6.68万人。成功承办第十五届省运会，芗剧《保婴记》荣获“五个一”工程奖。

各位代表，成绩的取得来之不易，得益于省委、省政府和市委的正确领导，得益于全市人民齐心协力、团结奋斗和各方面的大力支持。在此，我代表市人民政府，向全市人民，向人大代表、政协委员、各民主党派、工商联、各人民团体、无党派人士、离退休老同志和社会各界人士，向中央、省驻漳单位和驻漳部队、武警官兵、公安民警，向关心支持漳州发展的港澳同胞、台湾同胞、海外侨胞和国际友人，表示衷心的感谢！

在看到成绩的同时，我们也清醒地认识到发展中还存在不少困难和问题，主要是：经济下行压力持续加大，部分企业生产经营困难，市场需求不足，稳增长任务艰巨；大项目、好项目还不多，产业规模不大、结构不优、竞争力不强，龙头企业偏少，自主创新能力有待提高，新兴产业和现代服务业发展偏慢；新型城镇化滞后，中心城市辐射带动能力不强，农业农村基础还比较薄弱，脱贫攻坚任务仍然繁重；生态环境保护压力加大，节能减排面临新挑战；安全生产、食品药品监管、社会稳定等方面还存在不少隐患，特别是古雷“4·6”事故受损企业隐患整改、恢复生产任务繁重；教育、卫生、养老等民生欠账仍然较多；政府职能转变还不到位，不敢担当、慢作为、中梗阻和不贪不占也不为的问题仍不同程度存在，等等。我们一定要高度重视这些问题，采取更加有力措施认真加以解决。

二、精心实施“十三五”规划，全力加快建设富美漳州

根据《中共漳州市委关于制定漳州市国民经济和社会发展第十三个五年规划的建议》编制的《漳州市国民经济和社会发展第十三个五年规划纲要（草案）》，提出了今后五年经济社会发展的指导思想、目标任务和政策措施，全市地区生产总值年均增长10%左右，到2020年总量达4500亿元以上；一般公共预算总收入超过408亿元、地方一般公共预算收入超过259亿元；固定资产投资年均增长20%，城乡居民人均收入提前实现比2010年翻一番。

“十三五”时期我市国民经济和社会发展的指导思想是：高举中国特色社会主义伟大旗帜，全面贯彻党的十八大和十八届三中、四中、五中全会精神，以马克思列宁主义、毛泽东思想、邓小平理论、“三个代表”重要思想、科学发展观为指导，深入贯彻习近平总书记系列重要讲话精神，坚持全面建成小康社会、全面深化改革、全面依法治国、全面从严治党的战略布局，坚持发展第一要务，保持经济稳定较快增长，提高发展质量和效益，增强人民群众获得感。落实主体功能区规划，围绕“田园都市、生态之城”发展定位，着力创新发展、协调发展、绿色发展、开放发展、共享发展，全力打造四大经济增长极，深入实施“七个五”生态建设行动计划，加快形成适应经济发展新常态的体制机制和发展方式，全面推进经济建设、政治建设、文化建设、社会建设、生态文明建设和党的建设，全力推动富美漳州建设上新台阶。

（一）着力创新发展，加快产业转型升级。实施创新驱动战略、人才优先战略和质量强市战略，加大研发经费投入，全面提升自主创新能力。大力发展智能制造、绿色制造，加大智能制造的应用，推进产业技术向高端化、自动化、智能化发展。加速工业跨越发展，推动主导产业现代化、新兴产业规模化、传统产业高端化，力争到2020年四大主导产业产值突破7000亿元，四大战略性新兴产业产值达2720亿元。发展壮大海洋经济，建设海洋经济强市。加速发展互联网经济，培育新产业、新业态、新模式。提升发展现代服务业，推进生产性服务业专业化、生活性服务业精细化、旅游观光产业产品化、知识性服务业高端化，促进服务业发展提速、比重提升。加快发展特色现代农业，推动一产接二连三，加快产业融合发展，促进农业提质增效。全面深化改革，构建引领新常态的体制机制，完善要素市场体系，激发市场主体活力，营造有利于大众创业万众创新的良好环境。行政审批、对台合作、生态文明等领域改革走在全省前列。

（二）着力协调发展，推动总体趋于均衡。统筹城乡区域协调发展，优化空间布局，加快构建“一个中心、两大城镇组群、两大发展带、四大经济增长极”的区域新格局。大力实施“中心东移、跨江南扩、面海拓展”发展战略，彰显“绿城、花城、水城、历史文化名城”特色，做大做强做美中心城市。建设美丽乡村，推动城乡一体协调发展。实施重点突破、重点带动，加快建设产城融合的四大经济增长极，力争古雷用全市1%的土地创造50%的工业产值，工业区用全市5%的土地创造90%以上的工业产值。加快港口集疏运体系建设，完善市政设施，提高能源资源保障和防灾减灾能力。推进文化强市建设，促进物质文明和精神文明协调发展。

（三）着力绿色发展，建设生态文明先行示范区。以生态文明先行示范区和生态水系示范区建设为抓手，全面落实主体功能区规划，统筹各类空间性规划，坚守“三区五线”，构建美丽空间格局。深入实施“七个五”生态建设行动计划，加强环境保护和生态修复，开展大气、水、土壤污染防治行动，推进农村环境连片整治全覆盖，加快建设美丽漳州，让天更蓝、山更绿、水更清、城更美。健全生态文明制度体系，推进生态文化建设，倡导文明、绿色生活方式和消费模式。

（四）着力开放发展，争创对台对外合作新优势。丰富对台对外开放内涵，创建开放型经济新体制试点城市。以建设深化两岸产业合作示范区为基石，将漳州建成两岸人民深度融合示范区。发挥“海丝”重要节点城市的优势，努力将漳州打造成为闽南文化生态示范区、对台产业深度合作示范区和东盟港口城市产业合作试验区。深化与港澳的交流合作，提升漳港澳经贸合作层次。保护侨文化，做足侨文章，加强与侨界合作，加快侨区发展。坚持引进来和走出去并重、引资和引智并举，力争“十三五”期间实际利用外资55亿美元。推进厦漳泉大都市区同城化和闽粤经济合作区建设。

（五）着力共享发展，促进成果公平惠及人民。坚持人人参与、人人尽力、人人享有，不断提高人民群众的福祉和获得感。开展精准扶贫，坚决打赢脱贫攻坚战，确保2018年全面脱贫。推动更高质量就业，持续增加城乡居民收入，提前实现城乡居民人均收入比2010年翻一番。坚持优先发展教育，办好人民满意的教育。推进健康漳州建设，深化医药卫生体制改革，完善公共医疗卫生服务体系。大力发展社会救助和社会福利，积极应对人口老龄化，促进人口均衡发展。加强和创新社会治理，健全公共安全体系，推进法治漳州、平安漳州、诚信漳州建设。

三、开好局起好步，扎实做好2016年工作

按照市委要求，贯彻五大发展理念，坚持稳中求进工作总基调，以提高经济发展质量和效益为中心，以改革开放创新为动力，以加快产业转型升级为重点，在适度扩大总需求的同时，加强供给侧结构性改革，着力去产能、去库存、去杠杆、降成本、补短板，突出做好稳增长、调结构、强动力、惠民生、防风险，集中开展“项目建设年”“生态环境建设年”“营商环境建设年”活动，全力打好扶贫攻坚战，努力保持经济平稳较快发展，确保“十三五”发展开好局、起好步。

经济社会发展的主要预期目标是：全市生产总值增长10%，力争更快更好些，保持比全省高1.5个百分点左右的增幅；一般公共预算总收入增长5%，地方一般公共预算收入增长4%；全社会固定资产投资增长20%；外贸出口增长5%；实际利用外资增长7%；社会消费品零售总额增长11%，居民消费价格总水平涨幅控制在3%左右；城镇登记失业率控制在3.6%以内；城镇居民和农民人均可支配收入分别增长10%；落实节能减排降碳任务。

为实现以上目标，重点做好八个方面工作：

（一）扩大有效投资，全力打造四大经济增长极

加快重点项目建设。深化“项目建设年”活动,落实八大领域投资3000亿元以上。按照“两个50%以上”要求，每月开工一批亿元以上项目，其中，安排重点项目492个，年度投资1354亿元，力争市级重点项目新开工200个，建成或部分建成129个。围绕打造四大增长极，突出产业项目投资。举全市之力保障服务古雷发展，重点加快古雷炼化一体化项目开工建设和烯烃芳烃一体化、锦江石化芳烃己内酰胺一体化项目前期工作，确保腾龙化学苯酐等6个在建项目建成投产，争取PTA上半年、PX年底恢复试生产，完成古雷投资200亿元；更加注重招商选资，积极引进台湾四、五次加工项目、国内外专业中下游配套企业，推动荷兰皇家壳牌等项目早日落地，促进石化产业加快集聚、早出效益。厦门港南岸新城重点推进福欣特殊钢二期、龙海汽车产业园、金龙新能源汽车、正新橡胶二期、LNG接收站、双鱼岛等项目。漳州高新区重点推进绿宝生物科技园区、福马集团爱尚食品产业园等项目建设，加速形成高起点、高标准、规模化的产业园区。环东山岛经济区域重点推进旗滨玻璃、海洋生物科技园、漳州核电、大乌山景区等项目建设，加快建设闽粤经济合作区。

加大基础设施投入。着力构建更加完善的基础设施和配套体系，增强四大增长极吸纳要素、承载项目的能力。开工建设福厦（漳）铁路客运专线、厦漳城际轨道R3线、漳浦至古雷港城大道等项目，续建厦漳同城大道、沿海大通道、联六线、联十一线、厦蓉高速扩容工程、云平高速、漳州互通改造工程、漳江湾特大桥、诏安湾特大桥等重大交通设施，建成环东山岛路先导段、联十四线南靖东大路，实施古雷航道三期工程和南8#码头等5个港航项目，推进南靖至永定高速、厦门地铁角美延伸段等前期工作，加快构建互联互通、衔接紧密的港口集疏运体系。加快古雷新港城、管道燃气、污水处理等配套设施建设，实现古雷整岛封关运行。注重产城良性互动，加快完善教育、医疗、娱乐等生活配套，推进一批城市公共服务项目建设。

努力创新体制机制。加快申报设立古雷综合保税区、台商投资区保税物流中心（B型）、东山岛对台小额商品交易市场。深化审批制度改革，清理规范中介服务，全面实施清单管理，赋予四大增长极更多市级审批权限。深化“三比一看”，落实“一月一协调、一季一督查”工作机制。保障项目用地、用林、用海、用电等要素需求。完善政银企对接机制，推广PPP融资、债券融资等方式，带动更多社会资本参与投资。

（二）加速工业跨越，推动产业转型升级

做大做优增量。围绕提高供给体系质量和效率，落实《漳州市<中国制造2025>实施纲要》，实施产业龙头促进计划、新兴产业倍增计划和创新示范工程，开展制造业升级专项行动，培育壮大发展新动能。石化产业着重引办石化龙头，开展产业链招商，促进产业集聚。食品产业着重发展精深加工、专用型和功能性产品，抓好高新区食品产业园等项目。装备制造产业着重推广智能制造和应用，推动龙海汽车产业基地、诏安猛狮电动汽车整车生产基地、长泰雷天温斯顿锂电池项目投建投产。特殊钢铁产业着重发挥已有产能优势，促进产品升级和产业链条延伸,加快推进特殊钢产业园等项目。电子信息产业着重加快新一代信息技术推广应用，引进、培育光电龙头企业。建材产业着重提升行业技术水平，发展高附加值产品，推进东山玻璃及新材料产业园等项目。造纸及纸制品产业着重抓好联盛纸业等项目，引导现有企业技术改造，淘汰落后工艺与设备，原则上不再上新的纸浆项目。生物与新医药产业着重推动优势产品特色化、规模化发展，提高市场占有率，加快生物科技产业园建设。

改造提升存量。实施新一轮技改专项行动，完成技改投资540亿元以上。实施智能化改造提升工程和“机器换工”专项行动，推广智能制造应用，扶持做强一批重点智能制造企业，促进传统优势产业与高科技嫁接、与设计联姻、与品牌联动。强化创新对提高供给质量的支撑，实施“科技小巨人”培育计划，规划建设一批新型众创空间和孵化器，举办创新创业大赛，发展众创、众包、众扶、众筹支撑平台。落实省市工业园区扶持政策，设立工业园发展基金，推动园区优化布局、完善配套、提档升级。推动“大手拉小手”，促进专业分工，做大配套产业。实施中小企业成长计划，引导企业在漳州设立总部或注册法人公司。鼓励企业兼并重组，出清过剩产能。

促进产业融合。实施加快发展现代服务业行动，推动生产性服务业向专业化转变、向价值链高端延伸，提高生活性服务业品质和精细化水平。金融业重点强化对实体经济服务，制定地方金融与资本市场发展规划，设立市级产业股权投资引导基金，支持企业上市融资、再融资和债券融资，促进资产证券化；大力推进银团贷款，扩大信贷总量，防范金融风险。新增信贷规模300亿元以上、上市企业2家、场外市场挂牌20家。旅游业着力完善配套、整合资源、扩大宣传，打造集百里花卉走廊、海滨渔家风情、郊野生态休闲、古镇古村探秘、乡土民俗体验等为一体的生态文化旅游目的地，打响“清新福建·花样漳州”品牌，让“远方的客人留下来”，形成旅游消费。重点实施旅游景区创新提升三年行动计划和“三大工程”。互联网经济重点加快云计算、大数据、物联网等信息技术发展，推广一批“互联网+”项目，支持龙文、漳浦、云霄等重点电商园建设，整合提升电子商务聚集区，壮大电商经济规模。房地产业着重落实“去库存”政策，进一步打通商品房和安置房、保障房转换通道，提高棚改、征迁货币化安置比例，促进房地产市场健康发展。物流业着力加快漳龙物流园、五洲国际工业博览城等一批综合快递物流园区、配送中心和共同配送末端网店建设，加快发展冷链物流，支持第三方物流企业发展，促进各种运输方式无缝衔接。健康服务业着力降低门槛、放宽准入、扩大供给，大力发展健康养老服务、医疗保健、体育健身等业态。

（三）致力提质增效，发展特色现代农业

做精做强优势产业。在确保粮食面积176万亩、产量70万吨的基础上，更加注重效益和市场竞争力，突出品种、品质、品牌，做精做优九大特色优势产业，培育壮大林下经济。提升国家现代农业示范区建设水平，增强台湾农民创业园示范带动效应，推进24个特色产业“核心示范园”建设，新增设施农业2万亩。推进农业标准化生产，加强农产品质量安全认证和品牌培育，新增“三品一标”40个。大力发展农产品精深加工，扶持发展休闲观光体验农业，拓展农村电商，加快发展现代海洋渔业。

改善农业生产条件。抓好灌区续建配套和农田水利节水灌溉，开展农村饮水巩固提升工程。加快枋洋水利枢纽工程、龙海九九坑水库等水利项目建设，完成水利基础设施投资25亿元。推进信息化与农技推广有机融合，建立“五新”示范基地20个以上。抓好动植物重大疫病防治。加快气象现代化“六大系统”工程建设。加强水仙花基地保护和品牌开发。

创新农业经营方式。坚持和完善农村基本经营制度，全面推进农村土地承包经营权确权登记颁证，引导土地经营权依法规范有序流转，多种形式推进农业适度规模经营。实施新型职业农民素质提升工程，加快发展家庭农场、专业大户、龙头企业，引导农民合作社规范化建设，大力发展农机、植保等社会化服务组织。深化集体林权制度改革，稳步推进国有林场、供销社等改革发展。整合国有农业资产资源。创新农业农村金融服务，组建农业信贷担保机构，提升12个地方性“三农”综合保险项目覆盖面。落实第三次全国农业普查工作。

（四）加强城市工作，统筹城乡协调发展

进一步提升规划建设水平。强化规划引领，实行“多规合一”，加强城市设计。编制都市区综合交通、地下空间和人防建设、加油加气站布局、电动汽车充电设施等专项规划，完善高新区各项专项规划和市区北部片区规划，完成各县（市）城乡总体规划修编。协同推进厦漳泉大都市区同城化。围绕提高中心城市综合承载能力，实施新一批市政提升“五千工程”，更新改造雨、污、供水管网、城市道路各120公里、燃气管网80公里。加快推进南江滨路、东环城路、圆山大道、西环城延伸段等市区道路建设。大力推进“海绵城市”建设，完善一批水利排涝设施，建设西院、康山滞洪区。加大内河整治力度，加快内林、三湘江泵站建成投用，抓好圆山大道、北环城路10公里地下综合管廊试点。加快老旧社区燃气管网改造，市区力争改造通气1万户以上。发展城市综合交通，实施达达通公共自行车一期工程，新增更新公交120辆，新建停车楼5座，新增车位2800个以上。加快智慧城市国家试点建设，提升智能化管理水平。落实工程建设质量终身责任制，推动建筑产业现代化和绿色建筑发展。

打造宜居城乡环境。实施宜居环境建设项目800个，年度投资350亿元。加强城市精细化管理，巩固扩大全国文明城市创建成果，实施“十项改造提升工程”和“五项整治行动”，推动“垃圾不乱丢、车辆不违章、经营不占道”常态化。注重提升城市中心区绿化美化亮化水平，推进闽南文化生态产业走廊、闽南水乡、市区市民公园等项目。抓好县域交界处和“三边三节点”环境整治，完成绿道建设130公里。持续抓好美丽乡村建设，实施新一轮“千村整治、百村示范”工程，开展农村生活垃圾治理，确保所有乡镇建成1个农村生活垃圾转运设施，开展三级化粪池改造试点和村庄污水治理并逐步推开。

促进更多农民融入城镇。加快推进芗城、龙文全域城市化，东山、长泰两个县全域城乡一体化，建设角美、杜浔新市镇，提升发展大城关和一批特色中心镇，有序推进镇改街、村改居。深化户籍制度改革，全面实施居住证制度。探索建立农业转移人口市民化成本分担机制，推进基本公共服务向常住人口覆盖，把符合条件的转移人口纳入住房保障范围。深化土地制度改革，出台新的城镇户籍管理和基本公共服务配套政策，让进城的农民既保留享受农村承包地、宅基地、林地及农村集体资产收益分配等各种权益，又能享受城镇居民的就业、养老、医疗、教育、住房等公共服务。完善农民工职业技能提升计划，培育新型农民。

（五）激发活力动力，全面深化改革扩大开放

加大重点领域改革。围绕标准化、便利化、信息化，加快审批制度改革，简化投资审批程序，推进审批服务提速提效。深化工商登记制度改革，巩固扩大“三证合一”“一照一码”改革成果。加快推进事业单位分类改革。坚持放松审批、放宽领域、放活经营，完善国有资产监管制度，促进国有企业做大做强。支持民营资本以多种形式进入基础产业、社会事业以及特许经营领域，实施民企“回归工程”，释放民营经济活力。实施全面规范、公开透明的预算制度，优化财政支出结构，规范政府债务管理。推进公务用车制度改革，确保“三公”经费只减不增。规范招投标活动，坚决打击围标、串标、买标、卖标等违法行为。进一步抓好金融、价格、社保、社会事业等领域改革。

扩大对外开放格局。完善招商引资工作机制，强化“一把手”抓招商，突出靶向招商、以商引商、产业链招商，继续实行招商引资、项目对接考核。进一步复制推广自贸区经验，推进区域通关一体化。围绕打造海上丝绸之路先行示范区，传承月港辉煌，重点在经贸合作、人文交流、载体建设、产业融合、“海丝”遗址修复上有新进展。落实好稳出口各项政策措施，引导重点出口企业回归、货源回流。持续开展“漳州味·世界行·华夏行”系列行动，鼓励走出去开展国际化经营，提高产品市场占有率。

（六）发挥对台优势，促进漳台深度融合

推进产业深度对接。实施漳台产业对接升级计划，加快建设漳州深化两岸产业合作示范区和古雷深化两岸石化产业合作示范区，吸引台湾大型企业在漳设立区域性总部及服务外包基地。加强与台湾工商团体联系，深入对接百大企业、行业龙头和科技型中小企业，促成一批优势产业项目落地。加强关检合作，促进货物贸易和人员往来便利化。争取台资金融机构到漳设点，推动对台跨境人民币双向借贷等业务试点。持续深化漳台农业合作。

拓展文化社会交流。办好第八届海峡论坛相关活动。面向台湾“三中一青”，实施系列交流活动。创建台湾青年创业基地，吸引台湾青年来漳创业就业。加强涉台历史文化的抢救、保护、展示和传承。推动漳台乡镇、同名村、社区“点对点”共建，深化漳台姓氏宗亲、民间信仰、民俗文化交流。维护在漳台胞合法权益，鼓励参与社区管理。

（七）坚持生态优先，全面建设美丽漳州

深入实施“七个五”行动计划。深入开展“生态环境建设年”活动，巩固国家森林城市创建成果，完成造林绿化10.4万亩，力争通过国家生态示范区考核验收。把“七个五”生态建设行动计划具体化、项目化。“五海”保护重点抓好八尺门退堤还海工程、城洲岛生态修复等项目。“五沿”治理重点抓好植被保护和34个“青山挂白”治理项目。“五景”建设重点抓好闽南水乡建设、芝山公园二期等项目。“五行”管控重点抓好水源地保护、矿山生态环境恢复治理、生猪养殖标准化改造等项目。“五古”丰登重点抓好古城保护建设、江东桥周边环境整治、中山桥维修等项目。“五大”工程重点抓好沿海防护林体系工程、红树林湿地保护工程等项目。“五种”提倡重点抓好农村环境综合整治、绿色食品原料标准化生产基地、漳州南部垃圾焚烧发电厂等项目。

强力推进“三铁”治污。以“一库、两溪、三河”流域为主体，启动建设九龙江流域生态水系先行示范工程。开展为期三年的水污染专项整治，全面落实“河长制”，加大无证无照、偷排漏排污染企业、畜禽养殖面源污染、城中村污水整治力度。实施大气污染防治行动计划，开展施工、道路扬尘专项整治，巩固延伸胶合板污染企业治理，加大黄标车淘汰力度，持续改善空气质量。实施土壤污染防治，推进重金属、固体废弃物、持久性有机污染物治理，增强危险废物处置能力，实施农用地、建设用地分级分类管理，科学保护、利用和管控土地。

狠抓节能减排攻坚。严格落实节能减排责任制和环保监管“一岗双责”，实行能源和水资源消耗、建设用地等总量和强度双控。实施一批重点节能项目和减排工程，完成脱硫、脱硝设施升级改造。扩建芗城、云霄、诏安、平和县城污水处理厂，建设九龙江流域沿线一公里范围内重点乡镇污水处理厂，提高城乡生活污水处理水平。完善工业园区污水管网建设，实现全收集全处理。配套完善东墩、山格污水处理厂建设，确保正常运行。启动危废综合处置场和飞灰填埋场项目建设。加快淘汰落后产能，大力发展循环经济和清洁生产，推广节能环保技术应用，减少单位产出物质消耗。强化水资源“三条红线”管理，大力推广节水技术和产品。

（八）着力改善民生，让发展成果更多惠及人民

实施脱贫攻坚。把精准脱贫作为保障民生的首要任务，落实“三扶三到位”要求，健全“市级推动、县抓落实、工作到村、帮扶到户”的工作机制，实施精准扶贫打赢脱贫攻坚战三年行动，确保脱贫4万人以上，建设省市集中安置区（点）20个以上，完成造福工程搬迁5000户2万人以上。注重精准识别，规范建档立卡，确保贫困对象精准在册、到户到人。注重精准发力，分类施策，实行发展产业脱贫、转移就业脱贫、易地搬迁脱贫、发展教育脱贫、低保兜底脱贫、医疗保险和医疗救助脱贫，切实提高脱贫攻坚实效。注重精准管理，加强扶贫资金监管，强化年度考核评价，开展脱贫离任审计，建立贫困脱贫退出认定机制，确保脱贫责任落到实处。

健全社会保障体系。办好20件为民办实事项目。动工建设社会福利中心。加大就业创业扶持，新增城镇就业5万人，转移农村劳动力6.8万人。推进“三保合一”，推动城乡低保、农村“五保”扩面提标。落实基本医疗保险、大病保险先行报销要求。完善征迁补偿机制，健全被征地农民养老保障制度，帮助引导失地、失海农民二次就业创业。加快保障性安居工程建设和棚户区、预制板房改造，基本建成保障性安居工程6000套、新开工建设21828套。开工建设市区第二饮用水源，改造市区老旧供水管网。

重视教育文化体育事业。着力推动“义务教育发展基本均衡县”实现全覆盖；建设全市中小学发展项目17.3万平方米；实施县域内校长教师校际交流制度，加快农村薄弱学校改造；新建12所公办幼儿园，新增学位2500个，实现每个乡镇、街道至少建成一所独立设置的公办幼儿园;大力发展现代职业教育，推进市职教园区建设。实施免费特殊教育，重视发展终身教育和老年教育。加强教师队伍建设。推动民办义务教育健康有序发展。加强城乡公共文化服务体系和文化设施建设，重视历史文化名城、名镇、名村、名街、书院、文庙以及非物质文化遗产、传统艺术的保护，大力培养文艺人才，创作一批富有漳州特色的艺术精品。新建50个城市社区全民健身场所和工程点，创新体育场馆运营模式，扩大体育公共服务，积极发展体育健身产业。做好第二轮志书总编纂。推进县级国家综合档案馆建设。

加快建设健康漳州。加快推进市医院、市中医院高新区院区、古雷医院等项目建设，发展区域医疗中心，积极组建医疗联合体，新增床位1600张、卫技人员1000名。深化公立医院改革，推进公立医院人事与薪酬制度改革，完善药品集中采购和配送制度，鼓励医师多点执业，推动形成分级诊疗制度。加强基层医疗卫生机构建设，大力培养全科医生，支持乡村医生队伍发展。强化儿科、产科、精神卫生等薄弱学科建设。注重医养结合，建立医疗卫生机构和养老机构合作机制，培育引导社区养老。鼓励社会资本办医，力争非公立医疗机构床位数达到总量的20%以上。全面实施一对夫妇可生育两孩政策，促进人口长期均衡发展。

加强和创新社会治理。完善重大决策社会稳定风险评估机制，按照“路线图”和“七项机制”依法处理信访事项。加强平安漳州建设，完善立体化社会治安防控体系，严厉打击各类违法犯罪活动，严格落实各项反恐措施。完善城乡社区网格化服务管理，推进社区、社会组织、社工“三社联动”，强化社区自治和服务功能。严格落实安全生产“党政同责、一岗双责、失职追责”，全面执行建设项目安全设施“三同时”制度，推进安全生产标准化建设提升工程，开展道路交通安全综合整治三年提升工程，抓好火灾隐患、危爆物品、建筑施工等重点领域隐患排查整治。实施“食品放心工程”三年行动。深入实施“七五”普法。支持工会、共青团、妇联等人民团体发挥更大作用。扎实做好民族宗教工作。抓好新一轮全国“双拥模范城”创建工作，支持驻漳部队和武警部队建设，加强国防建设、国防教育、国防动员和人民防空，推动军民融合深度发展。

四、建设服务型政府，营造一流营商环境

经济进入新常态，政府要有新状态。深入践行“三严三实”，全面弘扬谷文昌精神，开展“营商环境建设年”活动，努力建设廉洁、勤政、务实、高效的服务型政府，着力营造一流的营商环境。

依法依规。政府一切权力来自人民、源自法授，必须依法全面履行政府职能。坚持法无授权不可为、法定职责必须为，推进依法决策、依法办事，自觉接受人大及其常委会监督、政协民主监督、社会监督和舆论监督，加强行政监察和审计监督。健全决策咨询机制，提高决策科学化水平。推进政务公开，让权力在阳光下运行。

利民惠民。民之所望，施政所向。坚持以人民为中心的发展理念，始终关注群众冷暖忧乐，眼睛向下、心装群众，努力保障好老百姓的票子、房子、孩子、身子和菜篮子，下大力气解决好空气环境、饮用水源和食品安全等问题，让老百姓吸进肺里、喝进嘴里、吃进胃里的都更洁净安全，努力提升人民群众的收入水平和生活质量，让群众有更多获得感、幸福感。

亲商重商。破除“熟人经济”，办事不管生人熟人一视同仁，本地外地不靠关系，审批做“减法”，服务做“加法”，多开“绿灯”、少亮“红灯”，多立路标、少设路障，多走“网”路、少跑马路，让企业办事更省时、省力、省钱、省心，做到企业赚钱、地方发展，营造亲商、爱商、尊商、重商的良好氛围。重承诺、守信用，讲规则、守规矩，构建责不缺位、权不越位、遇事换位、服务到位的新型政企关系，营造好的政策环境、市场环境、服务环境、社会环境、生态环境，让企业守法经营、安心发展、舒心生活。

严政廉政。严格落实党风廉政建设责任制，严格落实“八项规定”精神，严格执行《准则》《条例》。加强对土地出让、工程建设、产权交易、政府采购等关键环节的风险防范，用制度管人管事管钱。慎独慎微，始终不松防线、不碰红线、不越底线，严于律己，廉洁从政。

各位代表！目标催人奋进，发展全凭实干。让我们紧密团结在以习近平同志为总书记的党中央周围，在省委、省政府和市委的领导下，抓住机遇、开拓进取、凝心聚力、扎实工作，加快富美漳州建设，为建设机制活、产业优、百姓富、生态美的新福建，为全面建成小康社会作出新的更大贡献
